# Effect of Apical Third Enlargement to Different Preparation Sizes and Tapers on Postoperative Pain and Intra-canal Bacterial Reduction: A Randomized Clinical Trial

**DOI:** 10.4317/jced.63543

**Published:** 2026-05-29

**Authors:** Nermeen Awadallah Abbas, Alaa El-Din Hussein Diab, Manal Mohammed Abd-El Baky, Dina Ahmed Morsy

**Affiliations:** 1 PhD candidate, Department of Endodontics, Faculty of Dentistry, Cairo University, Egypt. Assistant Lecturer, Department of Endodontics, College of Oral and Dental Surgery, Misr University of Science and Technology, Egypt; 2 Professor, Department of Endodontics, Faculty of Dentistry, Cairo University, Egypt; 3 Associate professor, Department of Endodontics, College of Oral and Dental Surgery, Misr University of Science and Technology, Egypt; 4 Associate professor, Department of Endodontics, Faculty of Dentistry, Cairo University, Egypt

## Abstract

**Background:**

Apical preparation size plays a vital role in root canal instrumentation, yet the ideal size and taper remain a subject of debate. This randomized clinical trial was designed to assess the influence of different apical preparation sizes and tapers on postoperative pain and intracanal bacterial reduction.

**Materials and Methods:**

Fifty-six patients, diagnosed with necrotic mandibular premolars, were randomized into two groups according to apical enlargement size (#35 or #45). Each group was further divided by taper (0.04 or 0.06), resulting in four groups. Microbiological samples were collected before and after instrumentation, while postoperative pain was assessed at 6, 12, 24, 48, and 72 hours using the Numeric Rating Scale (NRS). Data analysis involved Kolmogorov-Smirnov and Shapiro-Wilk tests for normality, with Kruskal-Wallis, Friedman, and Fisher's exact tests; statistical significance was set as p &lt; 0.05.

**Results:**

At 6 and 12 hours, pain scores differed significantly between groups (p &lt; 0.05), with the highest levels at 6 hours followed by a significant decline by 12 hours (p &lt; 0.05). All treatment groups showed a marked reduction in bacterial counts after instrumentation (p &lt; 0.001). The percentage reduction varied significantly among groups (p &lt; 0.001).

**Conclusions:**

Larger apical preparations may induce early postoperative pain, yet improve intracanal bacterial reduction. Increased taper also contributes to enhanced bacterial reduction.

## Introduction

Postoperative pain represents a common and clinically significant complication associated with root canal therapy, which is largely attributed to periapical inflammation arising from residual intracanal microorganisms or the extrusion of debris and microbial irritants into periapical tissues during instrumentation (1). The principal purpose of root canal instrumentation is complete eradication of pathogens within the canal system along with the reduction of bacterial load to a level that facilitates peri-radicular tissues healing (2). Thorough debridement and disinfection of the apical third are essential, as this region -often referred to as the "critical zone"-commonly harbors residual microorganisms and introduces major challenges in endodontic treatment (2). Although root canal instrumentation is confined to the apical constriction, some degree of debris extrusion is unavoidable (3). The amount of extrusion varies with the instrumentation technique and is influenced by design features such as flute design, taper and alloy composition (3). Current evidence indicates that instrumentation kinematics does not exert a significant influence on postoperative pain (4). This suggests that postoperative pain is predominantly governed by multifactorial biological factors-most notably apical debris extrusion-beyond instrumentation type alone. Apical preparation size is a critical factor in root canal instrumentation, yet the ideal size and taper remain a debate, especially concerning their effects on postoperative pain, bacterial reduction, and dentine preservation (1 , 2 , 5). Consensus has not yet been established regarding the apical preparation size associated with the lowest incidence of postoperative pain (6). Few randomized clinical trials have examined the influence of apical enlargement to various sizes on either postoperative pain or intracanal bacterial reduction, and these parameters have been studied independently (6 - 9). Based on the available literature, no prior clinical trial has concurrently assessed the influence of apical enlargement size and preparation taper on both postoperative pain and intracanal bacterial reduction. Accordingly, this clinical trial was conducted to assess the influence of different apical preparation sizes as well as tapers on postoperative pain and intracanal bacterial reduction in mandibular premolars diagnosed with pulp necrosis. The null hypothesis stated that no significant differences would be observed among different apical sizes and tapers with respect to postoperative pain or the extent of intracanal bacterial reduction.

## Materials and Methods

- Study Design and Protocol Registration This study was designed as a single-center, prospective, parallel-group, randomized superiority trial with an allocation ratio of 1:1. The study protocol was prospectively registered on ClinicalTrials.gov (Identifier: NCT05620147). The trial was reported in accordance with CONSORT recommendations. - Ethics Approval and Consent The study protocol and informed consent documents obtained ethical approval from the Research Ethics Committee of the Faculty of Dentistry, Cairo University, ensuring compliance with ethical standards and human subject protection regulations (approval code: 3:1:23). The trial was carried out in accordance with the ethical principles outlined in the 2013 revision of the Declaration of Helsinki. Prior to enrollment, all participants were fully informed about the procedures, possible benefits, and risks and provided written consent to participate. - Sample size calculation Based on a previous study (6), the required sample size was determined under the assumption of normally distributed responses within each group with a standard deviation of 1.68, an estimated mean difference of 2, a level of 0.20 (power = 80%), and an level of 0.05. To minimize the effect of anticipated attrition, the calculated sample size was increased by 15%, yielding a final total of 56 participants, with 14 subjects allocated to each group. Sample size was calculated using G*Power software, version 3.1.9.4. Systemically healthy participants, classified as ASA I or II, aged between 18 and 40 years-regardless of sex-and diagnosed with asymptomatic pulp necrosis of mandibular premolars with a single root and single canal, with radiographic evidence of no periapical radiolucency corresponding to periapical index (PAI) score 1, were selected for the study (10). Participants were excluded if they had taken analgesics within the preceding 12 hours or presented with mandibular premolars that had been previously accessed. Teeth exhibiting active periodontal disease, extensive structural destruction, or associated with swelling, sinus tract, acute periapical abscess, acute exacerbation of a chronic abscess, or mobility of Grade II or III were also excluded. Randomization was performed by an independent investigator using a computer-generated sequence (http://www.random.org/) by an endodontist who was not involved in the trial, with allocation concealed in sealed opaque envelopes arranged in numerical order. These were opened by the operator only after access cavity preparation and enlargement with EdgeFile X7 to size 30/.04. While operator blinding was not possible, blinding was ensured for both the microbiologist conducting bacterial cultures and the statistician analyzing the data. Endodontic treatment was performed in one appointment. Local anesthesia was achieved by inferior alveolar nerve block with 1.8 mL of 2% mepivacaine (epinephrine 1:100,000) (Scandonest, Septodont, France). Cleaning of the operative field-including the tooth, rubber dam sheet, and clamp-was performed using 3% hydrogen peroxide, then, all surfaces were disinfected with a sterile cotton swab moistened with 5.25% sodium hypochlorite (NaOCl). Access cavity preparation was carried out using a sterile round bur size #2 and an Endo-Z bur (DENTSPLY, Tulsa Dental, TN, USA). Following access cavity preparation, the operative field and pulp chamber were disinfected with NaOCl and then neutralized with 5% sodium thiosulfate. Canal patency was confirmed using a stainless-steel hand K-file size #15 (K-FILES, MANI, INC., Industrial Park, Utsunomiya, Tochigi, Japan). Then, Pre-instrumentation samples (S1) were obtained by introducing brain heart infusion (BHI) broth (0.02 mL) into the canal using a sterile syringe (2). Three sterile paper points (#20/.02) were sequentially inserted until a level approximately 1 mm short of the radiographic apex, each maintained in position for one minute, then subsequently introduced into sterile tubes containing 1 mL of BHI broth for transport. Working length was established using an electronic apex locator (Root ZX; J. Morita USA, Irvine, CA, USA) and subsequently verified radiographically to be 0.5-1 mm off the radiographic apex. Canal shaping was performed using the EdgeFile X7 rotary system (EdgeEndo, Albuquerque, NM, USA) in the following sequence: #17/.04, #20/.04, #25/.04, and #30/.04. These instruments feature a triangular cross-sectional design and a variable helix angle and are manufactured using a proprietary heat-treated alloy (11). Instrumentation was performed using an endodontic motor (X-Smart; Dentsply Tulsa, DENTSPLY Maillefer, TN, USA) at a speed of 300 rpm and a torque of 2 N.cm (according to the manufacturer's instructions). Subsequently, participants were thereafter randomized into four groups, differing in their final apical enlargement size and taper: Group (35/.04): continued to 35/.04. Group (35/.06): continued to 35/.04 and 35/.06. Group (45/.04): continued to 35/.04, 40/.04 and 45/.04. Group (45/.06): continued to 35/.04, 40/.04, 45/.04 and 45/.06. After each file, irrigation was done, using 5 mL of 2.5% NaOCl for 1 minute, using a disposable plastic syringe equipped with a 30-gauge side-vented needle positioned 1 mm short of the working length. After dryness, canals were flushed with saline and rinsed with 5 ml of 5% sodium thiosulphate to eliminate any remaining NaOCl. After that, post-instrumentation samples (S2) were collected using the same protocol as S1, employing paper points corresponding to the final apical size. All samples were delivered to the microbiology laboratory within 20 minutes of collection. A final rinse of 17% EDTA was performed for 1 min, as a final flush, and separated from NaOCl with 5 ml of saline solution. The final apical preparation was verified by fitting master gutta-percha cones (EdgeFile, Gutta Percha Points; Albuquerque, New Mexico, USA) corresponding to the master apical file size and taper to the established working length. A periapical radiograph was obtained to confirm proper cone adaptation and length. After dryness, root canal obturation was then carried out using a modified single-cone technique, in which the corresponding master cone was used in combination with auxiliary cones (#25/.02), along with a resin-based root canal sealer (AdSeal, META BIOMED CO., LTD, Korea). Then access cavity was sealed temporarily using a provisional restorative material (Coltosol; Coltene/Whaledent, USA) with subsequent occlusal adjustment. The Numeric Rating Scale (NRS) (12) was used to record postoperative pain at 6, 12, 24, 48, and 72 hours. Patients were contacted at each interval to ensure compliance and monitoring. Definitive restorations were scheduled after 7 days. - Outcome measures Postoperative pain assessment Patients were asked to document both the occurrence and intensity of postoperative pain using NRS at 6, 12, 24, 48, and 72 hours after endodontic treatment, where 0 indicated no pain, 1-3 indicated mild pain, 4-6 indicated moderate pain, and 7-10 indicated severe pain. Microbiological analysis / Intra-canal bacterial count The intracanal bacterial load was quantified using a culture-based approach. Samples preserved in broth were vortexed for one minute and serially diluted ten-fold up to 10³ using brain heart infusion (BHI) broth. To enable the non-selective proliferation of both obligate and facultative anaerobes, 50 µL from each dilution was plated onto BHI agar enriched with 10% sterile defibrinated sheep blood, and the culture plates were incubated at 37°C for five days under an anaerobic environment generated by a GasPak system and verified with an anaerobic indicator. Following incubation, bacterial colonies were enumerated microscopically as colony-forming units per milliliter (CFU/mL), with adjustments made according to the respective dilution factors (13). - Statistical analysis Data normality was evaluated with the Kolmogorov-Smirnov and Shapiro-Wilk tests. Parametric data were analyzed using repeated-measures ANOVA with Bonferroni post hoc tests, while non-parametric data were evaluated with the Kruskal-Wallis and Friedman tests, followed by Dunn's post hoc test when applicable. Categorical variables were analyzed using Fisher's exact test or the Chi-square test. A significance level of p &lt; 0.05 was adopted, and analyses were conducted using IBM SPSS Statistics, version 25.0 (IBM Corp., Armonk, NY, USA).

## Results

**Figure 1:**
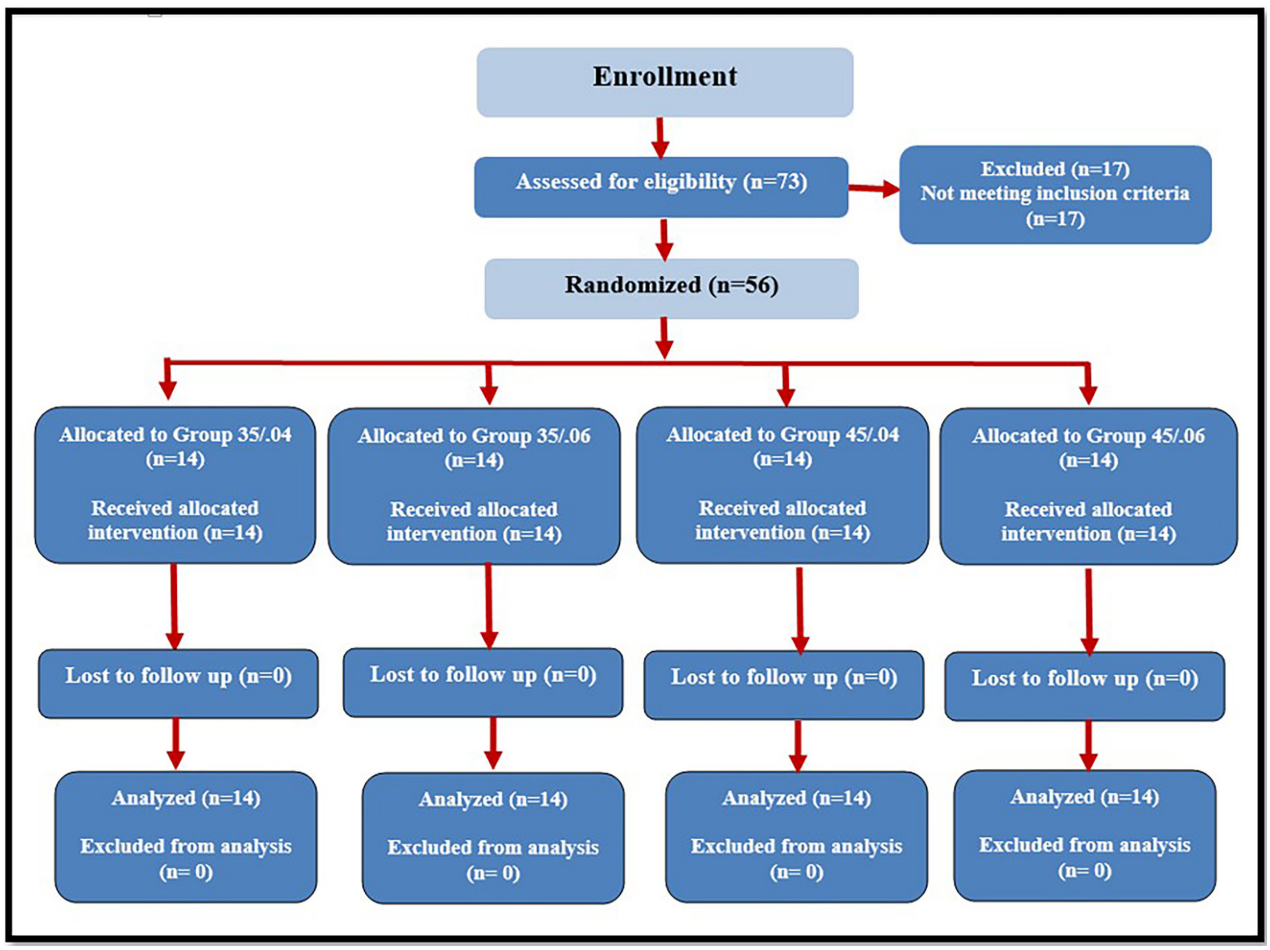
CONSORT flowchart showing the participants flow during different stages of the trial.

Participant retention was 100%, with all individuals completing the 72-hour follow-up and returning for definitive restoration. Recruitment was conducted between April 2023 and February 2025. The enrollment process and progression through study phases are presented in the CONSORT flowchart (Fig. 1).

1

Demographic data are detailed in Table 1.

Table 1

**Figure 2:**
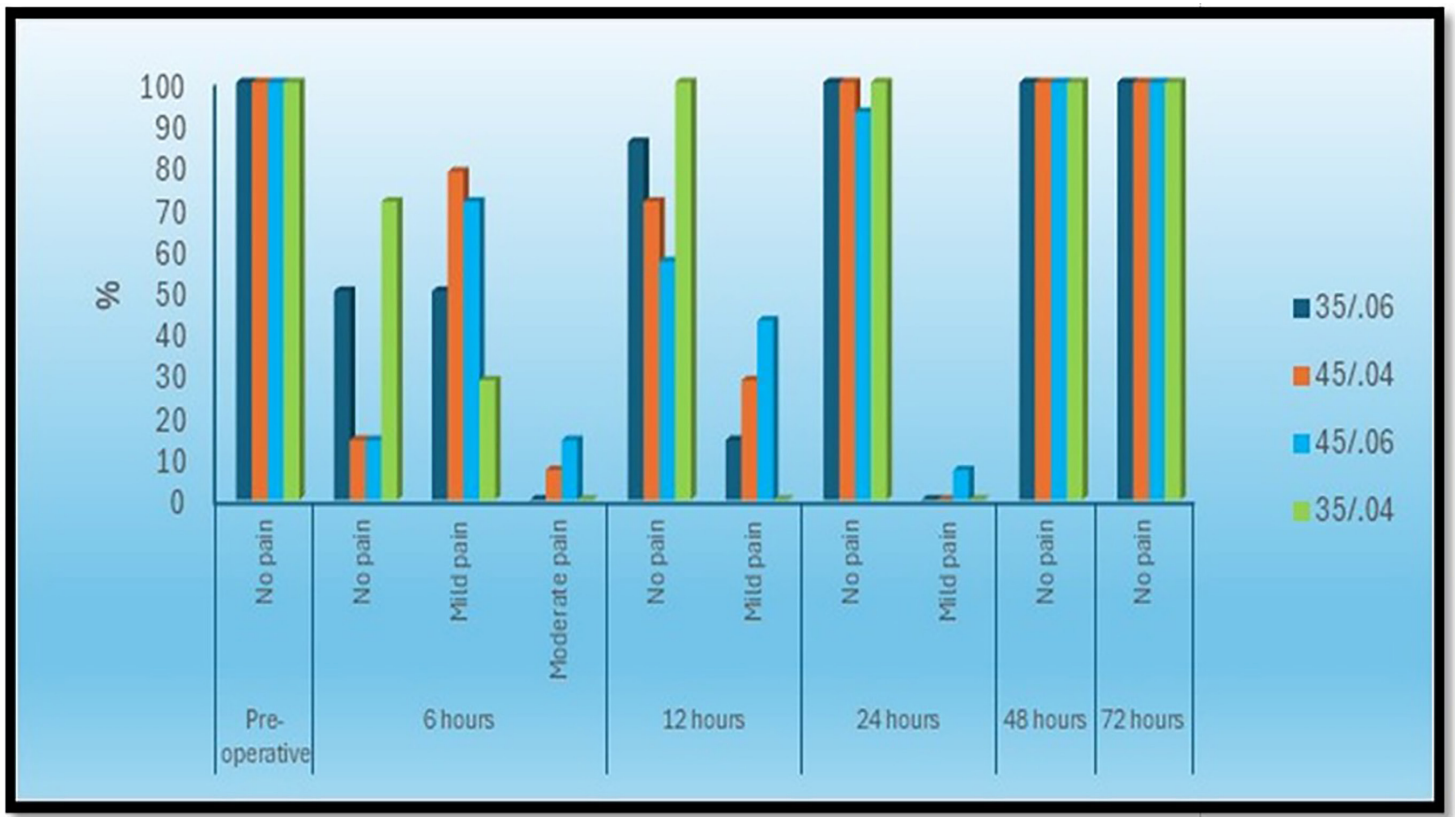
Bar chart representing intensity of pain among the four groups.

Intergroup analysis of pain intensity showed a significant difference at 6 h (p = 0.001), with no significant difference between Groups 45/.04 and 45/.06, or between Groups 35/.06 and 35/.04. At 12 h, Group 45/.06 had the highest pain score, significantly higher than Group 35/.04 (p = 0.035), but not significantly different from Groups 35/.06 or 45/.04. Intragroup analysis revealed a significant increase in pain scores from baseline to 6 h, followed by a significant decrease from 6 to 12 h. Pain incidence results are shown in Figure 2.

2

In Groups 35/.06 and 35/.04, pain intensity changed significantly over time (p &lt; 0.001, effect size = 0.286), with a rise in mild pain at 6 h followed by a decrease at 12 h; no pain was reported at later intervals. In Groups 45/.04 and 45/.06, pain intensity also changed significantly (p &lt; 0.001, effect size = 0.688), with increased mild-to-moderate pain at 6 h, decreasing progressively at 12 h and 24 h; no pain was reported at 48 h or 72 h. Results for bacterial reduction are displayed in Table 2.

Table 2

A significant difference in percentage reduction of both anaerobic and aerobic bacterial counts was evident among the four groups (p &lt; 0.001). Post hoc pairwise comparisons revealed that Group (45/.06) achieved the highest mean percentage reduction, while Group 35/.04 recorded the lowest for both bacterial types.

## Discussion

The current trial was conducted to investigate the influence of different apical sizes and tapers preparation on postoperative pain and intra-canal bacterial reduction in patients with mandibular premolars with necrotic pulp. Mandibular premolars, with a single root and canal, were chosen to be included in this study due to their relatively simple and consistent anatomy. Their straightforward canal configuration also allows for controlled assessment of apical and taper preparation effects on postoperative pain (14). Necrotic teeth were included because postoperative pain is more frequently observed in such cases, likely resulting from residual intracanal irritants (15). Their selection also eliminates preoperative pain as a confounding factor, allowing for a clearer evaluation of treatment-related pain (15). Since apical debris extrusion and over-preparation are major contributors to postoperative discomfort by disrupting the host-microbe balance, this sample was considered appropriate for evaluating the relationship between apical enlargement and postoperative pain (8). Additionally, only teeth without periapical lesions were included to avoid interference from chronic or extra-radicular infections, which may compromise the treatment outcome. In the present study, apical preparation sizes of #35 and #45 was selected, to evaluate their influence on postoperative pain and intracanal bacterial reduction in single-rooted and single canal mandibular premolars. Wolf et al. (16 - 18), using micro-computed tomographic analysis and three-dimensional imaging software, reported that the mean diameters of the physiological foramen in single-rooted, single-canal mandibular first and second premolars ranged from 0.28 to 0.37 mm and 0.28 to 0.40 mm, respectively. Suggesting that the final preparation size should be selected based on specific anatomical considerations, with a minimum apical size of ISO 30. Post-operative pain is a critical patient-related outcome that warrants careful examination in relation to various apical sizes and taper preparation in endodontic treatments (6). The findings of the present study revealed significant intergroup differences in postoperative pain were observed at 6 and 12 hours, with Group 45/.06 recording the highest scores, followed by Group 45/.04, while Group 35/.04 showed the lowest. Increased apical enlargement was correlated with greater pain during these time intervals; this could be attributed to the local inflammatory responses triggered by extruded bacteria and necrotic debris during instrumentation (19). The lack of significant differences beyond 12 hours suggests that this effect is transient and resolves relatively quickly. Moreover, the present study revealed that increasing the taper did not significantly increase postoperative pain incidence and intensity, potentially because increased taper improves irrigant penetration and canal debridement, which in turn reduces the risk of debris extrusion that consequently influences postoperative pain (1 , 20). Different findings were reported in a previous study (9), which demonstrated that greater apical enlargement resulted in significantly greater postoperative pain. The higher mean pain scores reported in that study, compared with the present findings, may be attributed to the inclusion of mandibular molars, where greater anatomical complexity could have influenced pain outcomes and contributed to the overall increased pain levels. However, another clinical study (6) evaluated different apical sizes and tapers, and reported no significant differences in postoperative pain between groups. The inconsistency with the present findings may be attributed to, that the treatment in that study was performed in two sessions, and the use of calcium hydroxide as an intracanal medicament between appointments may have alleviated postoperative symptoms. Pain scores of all four groups changed significantly over time, peaking at 6 hours and then declining. This pattern likely reflects the acute inflammatory response to tissue trauma during instrumentation, leading to the release of inflammatory mediators, which peaks within 4-6 hours (21). The apical preparation size plays a critical role in improving disinfection efficacy, and intracanal bacterial reduction is regarded as a meaningful surrogate endpoint due to its strong association with the healing of apical periodontitis (2). Significant intergroup differences were observed in the percentage reduction of both anaerobic and aerobic bacterial counts among the groups. Group 45/.06 demonstrated the highest mean percentage reduction, whereas Group 35/.04 exhibited the lowest mean percentage reduction in bacterial counts. These findings indicate that increasing both apical size and preparation taper enhances intracanal bacterial reduction. This can be attributed to larger apical preparations increasing the likelihood of instrumentation contacting a greater proportion of canal wall surfaces, thereby improving the removal of adherent biofilms and infected dentin (22 , 23). Increasing taper promotes more efficient irrigant flow, improving the removal of bacteria, necrotic tissue, and smear layer (20). Moreover, increased taper enhances irrigant flow at the apical third, as the needle can be introduced with less resistance at the middle-apical junction (20). The present results are consistent with the only available clinical trial (7) which demonstrated that larger apical enlargement significantly improved disinfection, irrespective of the irrigant used. This outcome is further supported by several in-vitro investigations, reporting that increasing apical preparation size enhances intracanal bacterial reduction in infected canals (24 - 27). However, an in-vitro study (28) reported that extensive dentin removal in the apical third is not essential as long as an appropriate taper is maintained, as this alone can facilitate effective irrigation. Consistent with our observations, additional in-vitro researches (29 , 30) have shown that greater taper improves canal cleanliness and enhances debridement. However, all of these aforementioned in-vitro results must be interpreted cautiously, as numerous biological and procedural variables present in in-vivo settings may not be replicated in laboratory settings. Despite its strengths, this study is subjected to certain limitations, including the comparatively small sample size and the use of a culture-based technique to measure intracanal bacterial reduction. For future research, it would be valuable to examine the correlation between apical enlargement-at varying preparation sizes and tapers- and microbiological culture findings in relation to periapical healing outcomes over long-term follow-up. Additionally, the use of intracanal medicaments, such as calcium hydroxide, may be considered, particularly in cases presenting with periapical lesions.

## Conclusions

Within the limitations of this trial, enlargement of the apical preparation size was linked with significantly greater postoperative pain during the early period (6, 12 h), suggesting that larger apical preparations may lead to greater initial periapical irritation. However, increasing apical preparation size enhances bacterial reduction in the root canal system. Preparation taper as well matters; a sufficient taper also improves intracanal bacterial reduction.

## Figures and Tables

**Table 1:** Descriptive statistics for baseline characteristics in the four groups.

Baseline characteristics	35/.06(n = 14)	45/.04(n = 14)	45/.06(n = 14)	35/.04(n = 14)	P- value
Gender [n, (%)]					
Male	8 (57.1%)	6 (42.9%)	6 (42.9%)	8 (57.1%)	0.767
Female	6 (42.9%)	8 (57.1%)	8 (57.1%)	6 (42.9%)	
Age in years [Mean, SD]	25.4 (5.7)	25.4 (3.3)	25.6 (4.9)	26.1 (5.3)	0.974
Tooth [n, (%)]					
First premolar	4 (28.6%)	6 (42.9%)	3 (21.4%)	6 (42.9%)	0.598
Second premolar	10 (71.4%)	8 (57.1%)	11 (78.6%)	8 (57.1%)	

1

**Table 2:** Descriptive statistics and results of the one-way ANOVA test for comparison between percentage reduction in bacterial counts among the four groups.

	35/.06(n = 14)		45/.04(n = 14)		45/.06(n = 14)		35/.04(n = 14)		P-value	Effect size(Eta Squared)
	Mean	SD	Mean	SD	Mean	SD	Mean	SD		
Anaerobic	87.49 B	1.04	86.52 C	1.08	89.28 A	1.37	86.15 C	0.84	<0.001*	0.703
Aerobic	87.48 B	1.77	88.5 B	1.91	90.52 A	1.7	86.09 C	1.8	<0.001*	0.466

*: Significant at P ≤ 0.05, different superscripts in the same row indicate statistically significant difference between groups
